# DWCox: A density-weighted Cox model for outlier-robust prediction of prostate cancer survival

**DOI:** 10.12688/f1000research.9434.1

**Published:** 2016-12-01

**Authors:** Jinfeng Xiao, Sheng Wang, Jingbo Shang, Henry Lin, Doris Xin, Xiang Ren, Jiawei Han, Jian Peng

**Affiliations:** 1 Department of Computer Science, University of Illinois at Urbana-Champaign, Urbana-Champaign, USA

**Keywords:** DREAM, Prostate cancer, Cox model

## Abstract

Reliable predictions on the risk and survival time of prostate cancer patients based on their clinical records can help guide their treatment and provide hints about the disease mechanism. The Cox regression is currently a commonly accepted approach for such tasks in clinical applications. More complex methods, like ensemble approaches, have the potential of reaching better prediction accuracy at the cost of increased training difficulty and worse result interpretability. Better performance on a specific data set may also be obtained by extensive manual exploration in the data space, but such developed models are subject to overfitting and usually not directly applicable to a different data set. We propose DWCox, a density-weighted Cox model that has improved robustness against outliers and thus can provide more accurate predictions of prostate cancer survival. DWCox assigns weights to the training data according to their local kernel density in the feature space, and incorporates those weights into the partial likelihood function. A linear regression is then used to predict the actual survival times from the predicted risks. In the 2015 Prostate Cancer DREAM Challenge, DWCox obtained the best average ranking in prediction accuracy on the risk and survival time. The success of DWCox is remarkable given that it is one of the smallest and most interpretable models submitted to the challenge. In simulations, DWCox performed consistently better than a standard Cox model when the training data contained many sparsely distributed outliers. Although developed for prostate cancer patients, DWCox can be easily re-trained and applied to other survival analysis problems. DWCox is implemented in R and can be downloaded from https://github.com/JinfengXiao/DWCox.

## Introduction

Prostate cancer is the 2nd leading cause of cancer death in men in the United States ^1^ and the 6th worldwide ^2^. In the past 10 years more than 2 million men in the US suffered from prostate cancer, and about 5% of those patients had metastatic castrate-resistant prostate cancer (mCRPC), an advanced form of the disease whose outcomes are poor and treatment remains unclear. Survival analysis based on clinical records has attracted researchers’ attention, since it can hopefully direct cancer treatment and help elucidate the disease mechanism.

The Cox regression ^3^, also known as the proportional hazards model, is a classic model in survival analysis. The simplicity and interpretability of the Cox model come from the proportional hazards assumption, which basically states that the risk can be estimated based on a linear combination of the predictive variables. A trained Cox model can calculate a relative risk score for a new patient based on his/her clinical information, and is thus able to rank patients with their expected order of death. It cannot, though, directly predict the expected time to death.

**Figure 1.**
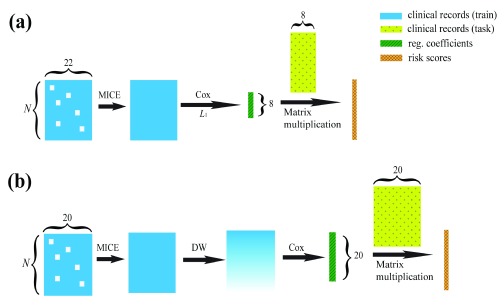
Illustration of how Halabi’s model (a) and DWCox (b) predict the risk scores. DWCox is also able to predict the days to death using linear regression with the risk scores (not demonstrated in this figure). *N*: number of patients. MICE: Multivariate Imputation by Chained Equations. *L*_1_: Lasso regularization. DW: Density-based weighting. Note that the objective functions in the Cox step of ( **a**) and ( **b**) are different, as discussed in the main text.

The Cox-based model proposed by Halabi *et al.* in 2014 ^4^ (referred to as **Halabi’s model** in the rest of this manuscript) is a state-of-the-art method for clinical prediction of prostate cancer survival. Halabi’s model is outlined in Figure 1(a). It starts with 22 features (“Halabi’s 22 features”), including some previously defined predictors of overall survival and some clinical parameters, picks out the eight most important features (“Halabi’s 8 features”) using *L*_1_ regularization, and predicts patients’ risks using those eight features only.

We propose **DWCox, a density- weighted Cox model** for predicting prostate cancer survival. DWCox was a best-performing method in the **2015 Prostate Cancer DREAM Challenge** ( **PCDC**), with performance better than or comparable to the best ensemble approaches. Simulations have shown that DWCox can achieve better performance than a standard Cox model when many sparsely distributed outliers exist in training data. DWCox is implemented in R in a way such that it can be easily re-trained and applied to other survival analysis problems, not restricted to prostate cancer. Please refer to the section “Data and software availability” for a download link and a citable link to the software.

## Methods

DWCox assigns weights to the training data according to their local kernel density in the feature space, and then trains an adopted Cox model with those weights incorporated into the loss function, as demonstrated in Figure 1(b). DWCox can also predict the actual survival time from the predicted risk score using a linear regression.

The development of DWCox underwent two phases. It was first developed and tested during the PCDC, and then further refined after its success. In this paper, unless something is stated to happen during the PCDC, DWCox should be understood as what it is now after the post-challenge refinements.

### Feature construction

Training DWCox requires a training group of *N* patients whose clinical features **X** and survival outcomes **Y** are known. **X** is an *N*-by- *M* matrix, where *M* is the number of clinical features and each element *X _ij_* is the value of the *j*th clinical feature of the *i*th patient. **Y** is an *N*-by-2 matrix, where each row gives the survival outcome of a patient. The 1st column of **Y** is a vector of the last observed survival time **t**, and the 2nd column is a vector of binary event indicators **d**. A patient *i* with *d _i_*= *TRUE* is known to die at time *t _i_*. Oppositely, one with *d _i_*= *FALSE* is known to be alive at time *t _i_*, but no information is available after *t _i_*. In the latter case, the record of that patient is said to be censored. In the data sets used in the PCDC, **Y** is known, while **X** needs to be constructed from clinical data.

To ensure fair comparison with Halabi’s model, DWCox constructed **X** in line with the way Halabi defined his 22 features, as summarized in Table 1 and described in details in the Supplementary material. Note that two features Halabi’s model started with, namely the Charlson comorbidity index and the Biopsy Gleason score, were not considered by DWCox since during the PCDC the former was not available in the training data and the latter was 100% missing in the leaderboard data. (Data were split into training, leaderboard and final validation sets. Details will be described in the Experiments section). That means *M* = 20.

**Table 1.** Clinical features used by DWCox, ordered with decreasing | *β _i_*|.

Variable name	Description	*β _i_*	In Halabi’s 8 features?
ast	aspartate aminotransferase level	0.567	
liver	liver metastases	0.497	
bmi	body mass index	-0.439	
alp	alkaline phosphatase level	0.260	Yes
ecogps	Eastern Cooperative Oncology Group performance status	0.204	Yes
alt	alanine transaminase level	-0.197	
race	race	-0.168	
hb	hemoglobin level	-0.117	Yes
lung	lung metastases	0.101	
analgesics	prior analgesics use	0.099	Yes
ds	disease site	-0.087	Yes
plt	platelet count	0.055	
psa	prostate-specific antigen level	0.050	Yes
wbc	white blood cell count	0.045	
bili	bilirubin level	0.041	
radio	prior radiotherapy	-0.040	
testo	testosterone level	0.038	
alb	albumin level	-0.027	Yes
ldh	lactate dehydrogenase level	-0.011	Yes
age	age	-0.008	

At this stage **X** was not complete (i.e. there were many missing elements in that matrix) due to missing information in the raw clinical records. Those missing values in **X** were imputed with the algorithm Multivariate Imputation by Chained Equations (MICE) ^5, 6^. The idea of MICE is to use Bayesian statistics to iteratively infer the missing values from other known and previously inferred values. Missing values in the training data were imputed with knowledge about the survival outcome, since it was argued that the outcomes could help generate less biased imputations ^7^. The survival outcome was incorporated into the imputation in the form of the Nelson–Aalen estimator as suggested by White and Royston ^8^. Imputation on the leaderboard and final validation data were done without using the survival outcome.

During the PCDC, three more binary features were used to indicate the trial ID (described in the Experiments section) of each patient. Those features were removed in post-challenge analysis so that the performance of DWCox does not depend on prior knowledge about the data source.

### Density-based weighting

After the imputation, the *N*-by- *M* matrix **X** can be represented by *N* points scattered in a *M*-dimensional space (“ **feature space**”). Each point represents a patient whose each coordinate is the value of one of his/her *M* clinical features. We assign each patient *i* a weight *w _i_* ∈ [0, 1] proportional to the estimated local Gaussian kernel density in the feature space. To calculate *w _i_*, we used the default settings of the function *kepdf* in the R package *pdfCluster* ^9^. These weights were then divided by the maximum value. Thus a patient with a higher weight indicates there are more other patients with similar clinical features.

### Model training

After density-based weighting, we used the R package *glmnet* ^10^ to maximize the weighted partial likelihood

$$L\left(\beta \right)=\prod_{i=1}^{m}\frac{e^{\sum_{j\in D_{i}}w_{j}\left({\text{X}}_{j}^{T}\beta \right)}}{{\left(\sum_{k:t_{k}\geq t_{i}}w_{k}e^{{\text{X}}_{k}^{T}\beta }\right)}^{\sum_{l\in D_{i}}w_{l}}}\;(1)$$

where ***β*** is a vector of the regression coefficients, **X**_*j*_ denotes the *j*th row of **X**, *t*_1_ < *t*_2_ < … < *t _i_* < … < *t _m_* is an increasing list of death times in **Y**, and *D _i_* is the set of patients died at time *t _i_*.

During the PCDC, *L*_2_ regularization was imposed to the objective function. The penalty weight was chosen to optimize the model performance (more specifically, iAUC, as defined in the next subsection) averaged over 100 repeated random sub-sampling validation on the training data. In each random sub-sampling validation experiment, 2/3 of all the training data were randomly selected to train the model with a wide range of possible penalty weights, and the iAUC was evaluated for each possible penalty weight on the remaining 1/3 of the training data. After the PCDC, the regularization was removed from DWCox since its contribution to the model performance was not obvious during the challenge and its removal sped up training.

After model training, the risk vector **r** of the training patients were calculated as

$$\text{r}=\text{X}\hat{\beta }.\;(2)$$

A higher risk indicates a shorter expected remaining lifetime for a patient. A linear regression $\text{t}=\hat{k}\text{r}+\hat{\text{b}}+\text{e}$ was then performed to correlate **t** to **r**, where $\hat{k}$ and $\hat{\text{b}}$ were the regression coefficients, and **e** was the error between the actual survival time and the estimated value (i.e. $t=\hat{t}+e$, where $\hat{t}=\hat{k}r+\hat{b}$).

### Prediction & evaluation

The trained model was used to predict the risk **r**_test_ and the remaining lifetime **t**_test_ for a new group of patients whose clinical features **X**_test_ could be constructed from clinical data while the outcome **Y**_test_ was not seen by the model. The model performance was then evaluated by comparing **r**_test_ and **t**_test_ to **Y**_test_.

The predicted risks **r**_test_ were evaluated with the integrated area under the ROC curve (iAUC) as described below. After obtaining $\hat{\beta }$ by maximizing Equation (1), we can estimate the risks of the patients ${\text{r}}_{\text{test}}={\text{X}}_{\text{test}}\hat{\beta }.$ Then an estimated order of death **ô** can be constructed by sorting **r**_test_ (i.e. **ô**_*i*_ = *j* where *i* = 1, 2, … , *N* and *r*_test, *i*_ is the *j*th smallest element of **r**_test_). By comparing **ô** with the actual outcome **Y**_test_, at any given time threshold *t _i_* we can calculate the area under the receiver operating characteristic curve AUC _*t*_*i*__. If we integrate AUC _*t*_*i*__ with respect to *t _i_* from the 6th to the 30th month, we get the integrated area under curve iAUC ∈ [0, 1]. The greater the iAUC, the better the predicted risks reflect the actual order of death.

DWCox also gives the estimated time to death of the test set: ${\hat{\text{t}}}_{\text{test}}=\hat{k}{\text{r}}_{\text{test}}+\hat{\text{b}}.$ In the PCDC ${\hat{\text{t}}}_{\text{test}}$ was evaluated by its RMSE from **t**_test_.

### Extended applications

The open-source release of DWCox is coded in a way such that it can be easily re-trained and applied to other survival analysis problems, not restricted to prostate cancer. To re-train and apply DWCox to a new dataset, users simply need to:

• Format their data into the three matrices **X**, **Y** and **X**_test_.
• Hit enter and get some coffee.
• Now they get the predicted risk **r**_test_ and time to event ${\hat{\text{t}}}_{\text{test}}$.

Here **X** and **X**_test_ can have as many rows (i.e. subjects) and columns (i.e. features) as needed. They can have missing values as well. More details can be found in the documentation inside the package.

## Experiments

### Challenge data & context

DWCox has been developed and evaluated with data from the comparator arms of four phase III clinical trials with over 2,000 mCRPC patients in total treated with first-line docetaxel. Those four trials and the corresponding data providers are:

• ASCENT-2 (Novacea, provided by Memorial Sloan Kettering Cancer Center) ^11^,
• MAINSAIL (Celgene) ^12^,
• VENICE (Sanofi) ^13^, and
• ENTHUSE-33 (AstraZeneca) ^14^.

During the PCDC those trials were referred to with their study IDs ( Table 2).

**Table 2.** Clinical trial used to develop DWCox.

Trial	Study ID	Number of patients	Survival outcome
ASCENT-2	ASCENT2	476	Known
MAINSAIL	CELGENE	526	Known
VENICE	EFC6546	598	Known
ENTHUSE-33	AZ	470	Hidden

The development and evaluation of DWCox began with the 2015 Prostate Cancer DREAM Challenge and continued after the challenge. The full anonymized information about the patients in trials ASCENT-2, MAINSAIL and VENICE was released to the challenge participants. As for trial ENTHUSE-33, the participants only knew the clinical records available at the beginning of the trial ("baseline clinical records"), while data obtained after the start of the trial including the survival outcome were visible only to the challenge organizers. The challenge goal was to develop models that used the baseline clinical records to predict the patients’ relative risk ( **sub-challenge 1a**), days till death ( **sub-challenge 1b**), and treatment discontinuation (sub-challenge 2) ( Table 3).

**Table 3.** Three sub-challenges of the PCDC.

Sub-challenge	What to predict	Evaluation metrics	Our participation
1a	Relative risk	iAUC	Yes
1b	Days to death	RMSE	Yes
2	Treatment discontinuation	(Irrelevant to this paper)	No

DWCox was trained on Trials ASCENT-2, MAINSAIL and VENICE (“ **PCDC training data**”) by the authors, and evaluated on Trial ENTHUSE-33 (“ **PCDC validation data**”) by the challenge organizers. Trial ENTHUSE-33 was further divided into a leaderboard set (157 patients) and a validation set (313 patients). The leaderboard set was used to run three leaderboard rounds. In each round, the challenge organizers randomly subsampled 80% patients from the leaderboard set, evaluated the participants’ models on that random sample, and returned the feedback to the participants. After the 3rd leaderboard round, each participating team submitted a final model, whose performance on the validation set was used to rank the teams. Bootstrapping was performed by the challenge organizers to make sure the winning teams gave statistically significantly better predictions than other teams and Halabi’s model. DWCox was involved in the leaderboard rounds of sub-challenge 1a and the final scoring round of sub-challenges 1a & 1b.

### Simulations

**Figure 2.**
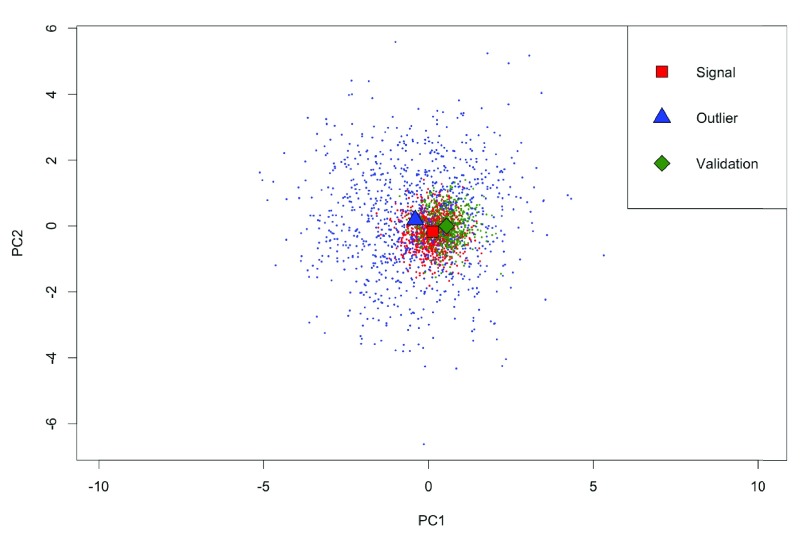
Scatter plot of the first two principle components of the signal, noise and validation groups in a simulated data set. Each point represents a patient. The shapes mark the mean of each group. (Best viewed in color).

Simulation experiments were performed to evaluate the contribution of density-based weighting to the model performance. DWCox was trained and evaluated on 100 simulated data sets (one example is given in Figure 2) separately, each of which was designed to mimic the real challenge data to some extent, while the randomness in the data generation process assured the variation across simulations. In each simulation, three groups of patients were simulated. Each patient had 20 features and an outcome.

One group (“ **signal group**”) represented a group of 1,000 patients that reflected the true correlation between the outcome and the features. The features were sampled from Gaussian distributions:

$${\text{X}}_{\text{signal},ij}∼\left(\mu_{j},\sigma_{j}^{2}\right)\;(3)$$

where *µ _j_* and *σ _j_* were the mean and standard deviation of the *j*th feature in the PCDC training data. Following the idea of R. Bender *et al.* ^15^, we simulated the survival time of each patient *i* with a Weibull distribution:

$$t_{\text{signal},i}={\left(-\frac{\text{log}\left(u_{\text{signal},i}\right)}{\lambda e^{{\text{X}}_{\text{signal},i*}^{T}\beta }}\right)}^{\frac{1}{v}}\;(4)$$

where *u*_signal, *i*_ ~ *U*(0, 1), *λ >* 0, *ν >* 0, and the subscript i* takes the *i*th row of the matrix. *U*( , ) denotes uniform distributions.

We would like to clarify a few things about Equation (4). Readers may get confused if they see an online manuscript with the same title and authors as those of Reference 15, where the minus sign of Equation (4) is outside the parenthesis. Obviously it is a typo, and it has been corrected in the version cited here. Although Equation (4) may not look like a Weibull distribution at first glance, the proof is a very straightforward and standard procedure. The shape and scale parameters of the Weibull distribution is *ν* and ${\left(\lambda e^{{\text{X}}_{\text{signal},i*}^{T}\beta }\right)}^{-1/\nu }$ respectively.

Such generated survival times follow a Cox model with the baseline hazard function *h*_0_( *t*) = *λνt* ^*ν*−1^ ^15^. The parameters *λ*, *ν* and ***β*** were estimated from the uncensored part of the PCDC training data as follows. First, we assumed ***β*** = **0** and fit a Weibull distribution to the distribution of **t**_uncensored_ to estimate *ν* and *λ*. Then DWCox was applied to the PCDC training data to obtain $\hat{\beta }$. At this stage $\hat{\beta }$ did not include ${\hat{\beta }}_{0}$, a constant term that affected $\hat{\text{t}}$ but not iAUC, since ${\hat{\beta }}_{0}$ played no role during the maximization process of Equation (1). We chose a ${\hat{\beta }}_{0}$ value such that the mean of the survival times simulated with Equation (4) was close to the mean of the uncensored survival times in the PCDC training data. After getting the estimates of *λ*, *ν* and ***β***, **t**_signal_ was simulated with Equation (4).

We then generated 1,000 more patients (“ **noise group**”) to represent outliers, or noises, in the training data. We made the outliers more sparsely and widely distributed in the feature space than the signal group by simulating

$${\text{X}}_{\text{noise},ij}∼(c_{1j}\mu_{j},{\left(c_{2j}\sigma_{j}\right)}^{2})\;(5)$$

where *c*_1 *j*_ ~ *U*(0.5, 1.5) and *c*_2 *j*_ ~ *U*(2, 4). In this section, identical mathematical symbols present in multiple equations (e.g. *µ*_*j*_ in Equation (3) and Equation (5)) share the same definitions and values.

The survival times of the noise group were simulated with a Weibull distribution independent of **X**_noise_:

$$t_{\text{noise},i}={\left(-\frac{\log(u_{\text{noise,}i})}{\lambda }\right)}^{\frac{1}{v}}\;(6)$$

where *u*_noise, *i*_ ~ *U*(0, 1).

A 3rd group of 500 patients (“ **validation group**”) was generated in a fashion similar to that of the signal group.

We let

$${\text{X}}_{\text{vali,}ij}∼(c_{3j}\mu_{j},{\left(c_{4j}\sigma_{j}\right)}^{2})\;(7)$$

where c _3 *j*_ ~ *U*(0.5, 1.5) and c _4 *j*_ ~ U(0.8, 1.2). The survival times are generated with

$$t_{\text{vali},i}={\left(-\frac{\log(u_{\text{vali,}i})}{\lambda e^{{\text{X}}_{\text{vali,}i*}^{T}\beta }}\right)}^{\frac{1}{v}}\;(8)$$

where *u*_vali, *i*_ ~ *U*(0, 1).

After simulating the three groups of patients, we mixed the signal and noise groups together to form a training set. DWCox and a 20-feature standard Cox model were trained on this training set, and evaluated with iAUC on the validation group.

## Results

DWCox was submitted to the sub-challenges 1a & 1b ( Table 3) of the 2015 Prostate Cancer DREAM Challenge. Sub-challenge 1a aimed at better predictions on the relative risks and order of death, evaluated with iAUC. Sub-challenge 1b evaluated the models using the RMSE between the predicted days to death and the actual time. While this manuscript is focused on our method, more details about other teams’ methods and performance can be found in papers from the challenge organizers and individual teams.

### Heterogeneity in the PCDC data

**Figure 3.**
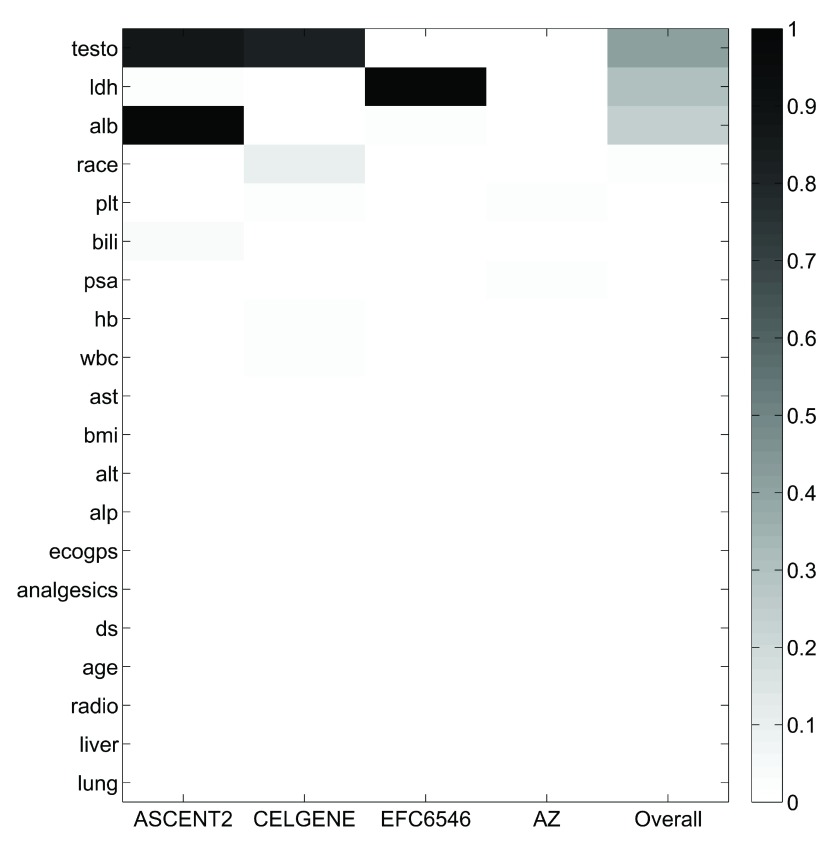
Heatmap of the percentage missing of the 20 clinical features used in DWCox.

**Figure 4.**
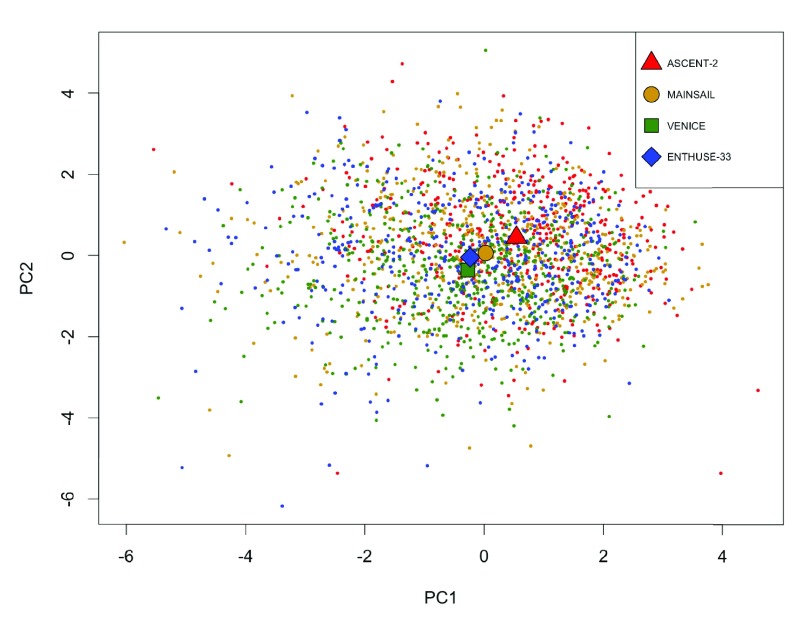
Scatter plot of the first two principle components of the four prostate cancer trials. Each point represents a patient. The shapes mark the average values of each trial.

Analysis of the PCDC data suggests that there exists rather high heterogeneity across the three training trials and the validation trial. The missing-rate profile of the 20 clinical features varies across trials ( Figure 3). The average values of the first two principle components of the 20 features of Trial ASCENT-2 is farther away from those of the validation trial, compared to those of the other two training trials ( Figure 4). Leave-one-trial-out cross-validation (i.e. to train with two training trials and evaluate with the left-out training trial) gives very different results when different trials are left out ( Table 4).

**Table 4.** Results of DWCox leave-one-trial-out cross-validation.

Left-out trial	iAUC
ASCENT-2	0.572
MAINSAIL	0.567
VENICE	0.685

Those facts give such a clue: If we consider the “true model” underlying the validation trial as the signal, it is very likely that the PCDC training data contain many outliers. Those outliers do not follow the “true model”, and thus tend to bring down the validation-set performance of models that failed to deal with the outliers properly during training. Therefore robustness against outliers is probably important to models aimed at winning the PCDC.

Indeed, several other winning teams of the PCDC tried hard to deal with the outliers in the training data. For example, the top performer (FIMM-UTU) of sub-challenge 1a decided to discard the entire ASCENT-2 trial, because after some manual exploration in the data space they found significant differences in clinical variables that set this trial apart from the other trials. Our team (Team Cornfield) instead used all available data and let DWCox automatically handle the outliers.

### Results on the PCDC data

**Figure 5.**
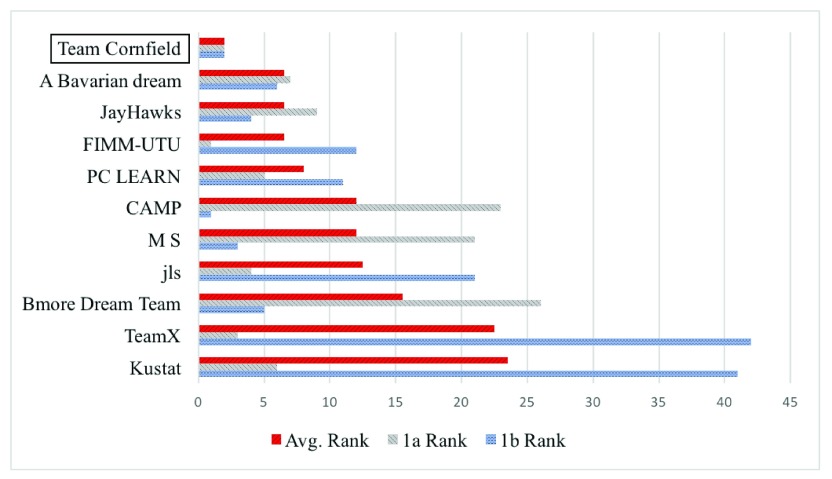
Ranking of the top teams in sub-challenges 1a & 1b. The six best teams of each sub-challenge are included. DWCox was submitted by the authors’ Team Cornfield.

DWCox obtained the best average ranking in sub-challenges 1a & 1b among about 50 models ( Figure 5). On the PCDC validation data, DWCox gave an iAUC of 0.7789 and a RMSE of 194.8650 days, out-performing Halabi’s model which gave an iAUC of 0.7581 and a RMSE of 196.6704 days. Bootstrapping has shown that DWCox outperforms Halabi’s model with a Bayes Factor (BF) *>* 3. Note that while the other numbers in this paragraph are official results provided by the challenge organizers, the Halabi RMSE is not. In order to get the Halabi RMSE, we implemented a Halabi’s model and appended to it a linear regression step similar to the one in DWCox. After applying bootstrapping and the BF *>* 3 threshold against other teams’ submissions, the challenge organizers reported DWCox as a winner in sub-challenge 1b and a runner-up in sub-challenge 1a. The winner of sub-challenge 1a, FIMM-UTU, obtained an iAUC of 0.7915 and a RMSE of 201.3779 days. Their model is an ensemble of penalized cox regressions developed with extensive manual exploration in the data space. More details about the challenge results can be found at https://www.synapse.org/#!Synapse:syn2813558/wiki/232674. Table 1 gives the regression coefficients determined by DWCox.

**Figure 6.**
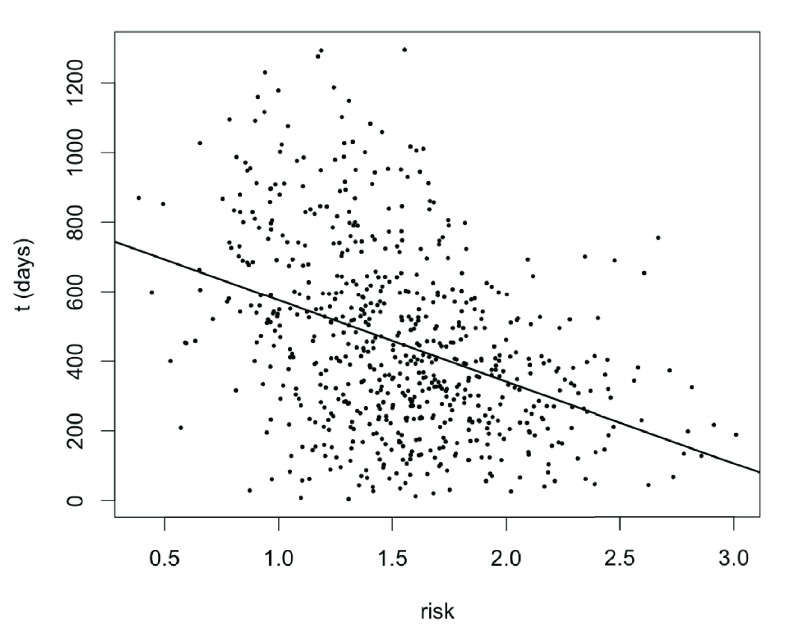
Scatter plot of the uncensored survival time versus the predicted risk on the PCDC training data. The straight line is the linear regression line with slope = -234.6, intercept = 810.3 and adjusted *R* ^2^ = 0.1513.

An inverse correlation between the actual survival time **t** and risk scores **r** was observed ( Figure 6). Note that the adjusted *R* ^2^ of the linear regression $\hat{t}=\hat{k}r+\hat{b}$ is small (0.1513), and the shape of the **t** vs **r** plot implies that there may exist models better than a linear regression for capturing their correlation.

### Results on simulated data

**Figure 7.**
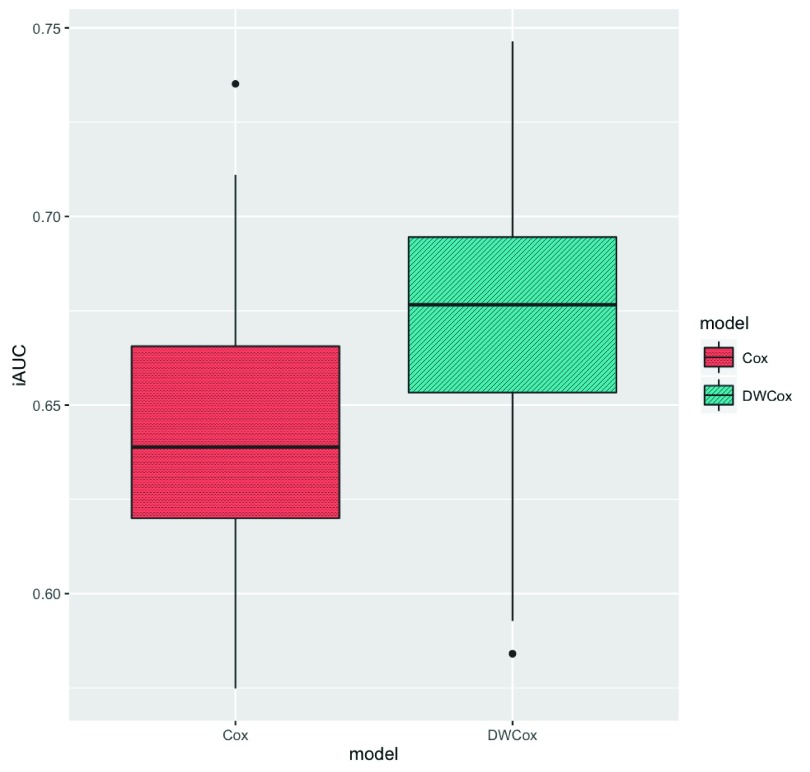
Boxplot of the iAUC of DWCox and a standard Cox model in 100 simulations. The boxes show the medians and inter-quartile ranges (IQR). The vertical black lines extends from the boxes by at most 1.5 IQR. Black points represent experiments whose iAUC is more than 1.5 IQR away from the boxes.

**Figure 8.**
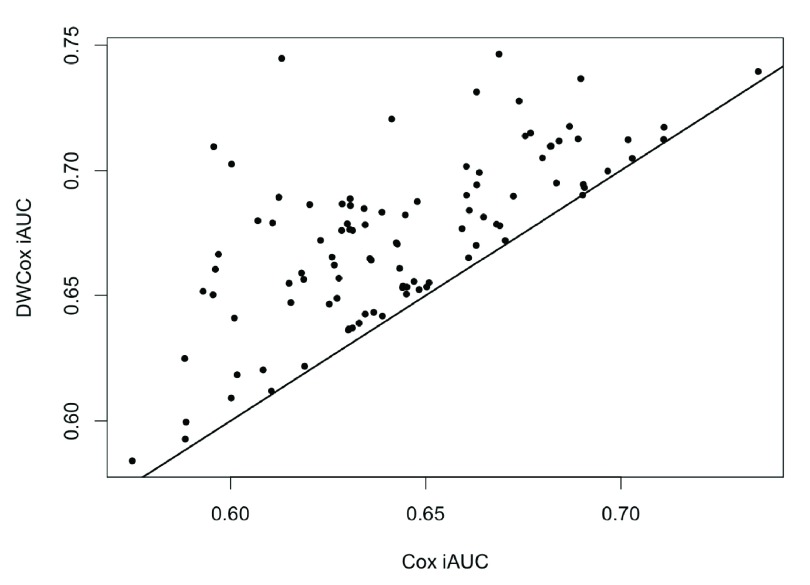
DWCox iAUC vs the standard Cox iAUC in 100 simulations. Each point is given by a simulation. The straight line has slope = 1 and intercept = 0.

In the 100 repeated simulations (described in the Experiments section), DWCox performed better than a standard Cox model when as many as half of the training data were outliers. DWCox not only gave better average performance over the 100 experiments ( Table 5, Figure 7), but also performed consistently better in each experiment ( Figure 8, paired t-test p-value = 2.1 *×* 10 ^−20^). The improvement in performance clearly resulted from the density-based weighting, since everything else was the same across the two models.

**Table 5.** iAUC statistics of 100 simulations.

	DWCox	Cox
Mean(iAUC)	0.674	0.643
SD(iAUC)	0.033	0.033

Note that in the simulations we used iAUC but not the RMSE to evaluate model performance. There are three reasons for that. 1. iAUC evaluates model performance on the validation data in a more comprehensive manner, while RMSE is based on individual predictions which are independent of each other. 2. DWCox’s time-to-event prediction is dependent on its predicted risks. 3. A standard Cox model does not directly give the predicted time-to-event.

## Discussion

We propose DWCox, a density-weighted Cox model for survival analysis that is more robust against overfitting outliers from the training data. In our simulations DWCox outperformed the standard Cox when as many as half of the training data were noise. In the 2015 Prostate Cancer DREAM Challenge (the PCDC), DWCox obtained the best average ranking in sub-challenge 1, which was to predict the risk and survival time of prostate cancer patients from clinical data available at the beginning of trials.

DWCox was one of the only two models among the seven winners of the PCDC sub-challenge 1 that did not use super-learners (or ensemble methods). (The other model ^16^ of the two was a standard Cox trained with different features. In Figure 5 the corresponding team name is M S.) This is a remarkable achievement, since super-learners usually give better results than single methods. Given that now DWCox gives results comparable to or better than ensemble methods, there are even more reasons to prefer DWCox over ensemble ones in real-world applications. During the training of ensemble methods, there often exist some empirical parameters (e.g. the number of base learners to use) that require more hyperparameter tuning, because people do not know exactly which value works best and why. In addition, some ensemble methods (e.g. random forests) have great built-in randomness and produce very complex models, and thus it is sometimes hard to interpret and understand the results they give. Oppositely, the training phase of DWCox involves no empirical parameters or built-in randomness (except when the user wants DWCox to impute the missing data with MICE), and the results can be easily interpreted.

DWCox’s success in the PCDC should be credited to its density-based weighting mechanism. There exists inter-trial heterogeneity in the PCDC data, which implies some training trials may contribute more signals than others, while some may contain more outliers. It turned out that several top-performing methods of the PCDC recognized such problem and tried to handle it properly. DWCox achieved this by taking in all training data and automatically weighting away outliers with the local Gaussian kernel density. DWCox can be easily re-trained and applied to other data sets, not restricted to prostate cancer survival data.

Perhaps the greatest limitation of DWCox also lies in its density-based weighting mechanism. Such mechanism cannot weight away outliers falling inside the signaling region of the feature space, or outliers that happen to cluster together in the feature space and thus give a local kernel density similar to those of the signals. In another extreme case where the data contain few outliers and follow a standard Cox model rather well, introducing weights into the partial likelihood function can make the performance worse. Therefore it is better to apply DWCox to cases where the data are expected to contain some sparsely distributed outliers.

## Data and software availability

This publication is based on research using information obtained from www.projectdatasphere.org, which is maintained by Project Data Sphere, LLC. Neither Project Data Sphere, LLC nor the owner(s) of any information from the web site have contributed to, approved or are in any way responsible for the contents of this publication.

The clinical trial data used in the PCDC, in its raw and processed format, can be accessed at: https://www.projectdatasphere.org/projectdatasphere/html/content/149?pcdc=true. Challenge documentation, including the detailed description of the Challenge design, overall results, scoring scripts, and the clinical trials data dictionary can be found at: https://www.synapse.org/ProstateCancerChallenge.

An R implementation of DWCox can be downloaded from https://github.com/JinfengXiao/DWCox. A citable snapshot of that GitHub repository has also been archived with the DOI: 10.5281/zenodo.167143 ^17^.

## Supplementary material

This section gives details about the 20 predictors used by DWCox in the PCDC.

• age: Categorical variable with 3 levels. 18–64 years old = 1; 65–74 years old = 2; at least 75 years old = 3.
• alb: Albumin level in g/L. Continuous variable.
• alp: Natural logarithm of the alkaline phosphatase level in U/L. Continuous variable.
• alt: Natural logarithm of the alanine transaminase level in U/L. Continuous variable.
• analgesics: Prior analgesics use. Binary variable. 1 means yes; 0 means no. Note that this is not exactly the "opioid analgesic use" as appeared in the baseline paper, since the latter is not contained in the challenge data set.
• ast: Natural logarithm of the aspartate aminotransferase level in U/L. Continuous variable.
• bili: Natural logarithm of the total bilirubin level in µmol/L. Continuous variable.
• bmi: Natural logarithm of the body mass index in kg/m ^2^. Continuous variable.
• ds: Disease site. Categorical variable with 3 levels. 0 means the disease sites are not at bones or viscera. 1 means the disease sites are at bones but not at viscera. 2 means at least some disease sites are at viscera.
• ecogps: Eastern Cooperative Oncology Group performance status. Categorical variable with 3 levels (0, 1 and 2). The greater the value is, the more severe the situation is for the patient. Technically this variable should have 6 levels (0, 1, …, 5), but Halabi’s model only considers the first 3 levels. Besides, in the challenge training data there is only 1 patient whose ecogps is greater than 2 (and it is 3). Therefore DWCox sets all ecogps > 2 to 2.
• hb: Hemoglobin level in g/dL. Continuous variable.
• ldh: Lactate dehydrogenase level. Binary variable. 1 means the lactate dehydrogenase level is greater than 200 units/liter, which is considered as the value of the upper limit of normal (ULN) ^18^. 0 means the opposite.
• liver: Liver metastases. Binary variable. Yes = 1; No = 0.
• lung: Lung metastases. Binary variable. Yes = 1; No = 0.
• plt: Natural logarithm of the platelet count in 10 ^9^/L. Continuous variable.
• psa: Natural logarithm of the prostate-specific antigen level in ng/mL. Continuous variable. The reason for taking logarithm is to make the distribution less skewed.
• race: categorical variable with 4 levels. White = 1; Asian = 2; Black = 3; Other or Hispanic = 4.
• radio: Prior radiotherapy. Binary variable. Yes = 1; No = 0.
• testo: Testosterone level in nmol/L. Continuous variable.
• wbc: Natural logarithm of the white blood cell count in 10 ^9^/L. Continuous variable.
