# Supplementary material for: DWCox: A density-weighted Cox model for outlier-robust prediction of prostate cancer survival
Source: F1000Res. 2016 Dec 1;5:2806. [Version 1] doi: 10.12688/f1000research.9434.1 (PMC5321125; doi:10.12688/f1000research.9434.1)
Supplement: Peer review history

# Peer review: Referee response for version 1

**DOI:** 10.5256/f1000research.10162.r20019

**Published:** 2017-02-17

**Authors:** Riku Klen, Mehrad Mahmoudian

**Affiliations:** 1 University of Turku, Department of Mathematics and Statistics, Turku, Finland; 2 Turku Centre for Biotechnology, University of Turku, Turku, Finland

General comments

 The article introduces a density-weighted Cox model (DWCox). The model was created by Team Cornfield in the 2015 Prostate Cancer DREAM Challenge for outlier-robust prediction of survival. The article is well written and the introduced method is novel. The only major comment about the article is that the comparison of the new method and the existing methods could be more complete.

 The authors test the DWCox method with the 2015 Prostate Cancer DREAM Challenge data and simulated data. They compare DWCox method with the Cox model and Halabi’s model introduced in reference 4. I suggest that the authors also consider as a fourth alternative method the Smaletz method ^1^ due to the fact that it was also used in Halabi et al. article. To make the study complete these 4 methods should be compared and the results reported for the simulated data and the 2015 Prostate Cancer DREAM Challenge. It would be interesting to see complete results in the spirit of Table 5 and Figure 7.

 Table 1 shows the 22 featured ranked by DWCox. It would be interesting to know how DWCox behaves when the features with missing values (namely race, testo, ldh or alb) were omitted. Based on the Table 1 these features have small weight and they might have little effect on the prediction results.

 Additionally, it would be interesting to see how the method behaves on other data sets. However, this general study might be out of the scope of this article.

 Furthermore, it would be more descriptive to explain how the missing values are handled in DWCox approach in page 5.

 Detailed comments:

 - page 1, line 6 of abstract: The result will not be worse in interpretation, but the model’s interpretability will decrease. Hence the last word “result” should be substituted by “model”

 - page 3, line 18: It should be specified that the reason Cox is not appropriate for testing time dependency is due to the nature of semi-parametric models that are have no assumption on the shape of the hazard function. ^2^

 - page 3, line 30: It would be nice to have a citation to clarify the statement thatDWCox was performing “better than or comparable to the best ensemble approaches”

 - page 10, line 3: the comparison represented in Figure 8 was done using t-test. Maybe Wilcoxon test would have been more appropriate.

We have read this submission. We believe that we have an appropriate level of expertise to confirm that it is of an acceptable scientific standard, however we have significant reservations, as outlined above.

## References

[1] SmaletzOScherHISmallEJVerbelDAMcMillanAReganKKellyWKKattanMW: Nomogram for overall survival of patients with progressive metastatic prostate cancer after castration. *J Clin Oncol*.2002;20(19) : 10.1200/JCO.2002.11.021 3972-82 10.1200/JCO.2002.11.021 12351594

[2] GarsonGD: Paremetric survival analysis. *Statistical associates blue book series 17*.2012;

# Peer review: Referee response for version 1

**DOI:** 10.5256/f1000research.10162.r20018

**Published:** 2017-02-13

**Authors:** Sebastian Pölsterl

**Affiliations:** 1 Insitute for Cancer Research , London, UK

The authors present an interesting extension of the well-known Cox proportional hazards model if data contain outliers. They demonstrate the advantage over the traditional Cox model on synthetic data and applied their proposed model to a real world problem in the context of the 2015 Prostate Cancer DREAM challenge.

**Major issues**

 Unfortunately, the author only provides little insight in the motivation for choosing a kernel density estimator to determine the sample weights. In particular, traditional kernel density estimation is only applicable to continuous random variables, whereas feature vectors comprised of clinical variables can contain continuous as well as categorical variables. It is unclear how density estimation was performed when feature vectors are a mix of continuous and categorical variables. Moreover, I strongly suggest to explicitly mention the assumption of the proposed density-weighted Cox model. The authors state that their proposed model is suitable when data “contain some sparsely distributed outliers.” A more systematic approach to thoroughly formulate this assumption would be highly appreciated.

**Minor issues**

1. Page 2, paragraph 2:
  1. Reference 3, please cite the original paper Cox 1972.
2. Page 4, paragraph 3:
  1. I would suggest to change reference 5 to the original work on multiple imputation by Rubin: D. B. Rubin, Multiple imputation for nonresponse in surveys, John Wiley & Sons Inc., 1987. ^1^
3. Page 5, paragraph 2:
  1. How were the candidate values for the L2 penalty chosen?
4. Page 5, paragraph 3:
  1. It is not clear what the coefficients \hat{b} represent. It seems they are not associated with any features, only \hat{k} is.
  2. Is the error e assumed to be normally distributed? If yes, such a choice might be problematic, because survival times usually follow a skewed distribution. Representing the log survival time as a linear model, as in the case of the accelerated failure time model, is usually preferred.
5. Page 5, paragraph 5:
  1. Please cite the original work on iAUC:
    - H. Hung and C. T. Chiang, “Estimation methods for time-dependent AUC models with survival data,” Canadian Journal of Statistics, vol. 38, no. 1, pp. 8–26, 2010. ^2^
    - H. Uno, T. Cai, L. Tian, and L. J. Wei, “Evaluating prediction rules for t-year survivors with censored regression models,” Journal of the American Statistical Association, vol. 102, pp. 527–537, 2007 ^3^
  2. It should be mentioned that the RMSE used in the challenge was only with respect to uncensored survival times in the test set.
6. Page 5, extended applications:
  1. I would suggest to remove this short section, because it is already clear from the description in the text, that the author’s propose a general model that can be applied to any survival data.
7. Page 7, paragraph 2:
  1. Please cite the recently published paper summarising the Prostate Cancer DREAM challenge
8. Page 7, Results on the PCDC data:
  1. The author’s stated earlier that the Halabi model is based on a Cox model and that a Cox model is not able to directly predict time to death. However, the authors mention that the Halabi model achieved an RMSE of 196.6704. How was this value obtained, if the model is not applicable for this task?
  2. It would be helpful of the authors could add the exact Bayes factor of the proposed model.
9. Page 9, Results on simulate data
  1. To better understand the benefit of the proposed density-weighted Cox model, it might be interesting to plot the the i-th weight against the i-th residual in the unweighted Cox model. I would assume that samples with high residuals are assigned a low weight, leading to an overall better prediction.

**Grammar**

 The text contains several grammatical errors and convoluted formulations, which damps the overall presentation. I strongly suggest to improve the grammar and wording. I’m only highlighting some obvious errors below.

 Page 3, paragraph 2:

- The simplicity and interpretability of the Cox model come from the proportional hazards assumption

Page 4, paragraph 5:

- Each point represents a patient whose each coordinate is the value of one of his/her *M* clinical features.

Page 6, paragraph 1:

- The challenge goal was to develop models

Page 7, paragraph 1:

- The shape and scale parameters of the Weibull distribution is

I have read this submission. I believe that I have an appropriate level of expertise to confirm that it is of an acceptable scientific standard, however I have significant reservations, as outlined above.

## References

[1] RubinDB: Multiple Imputation for Nonresponse in Surveys. *John Wiley and Sons, Inc*.1987; 10.1002/9780470316696 10.1002/9780470316696

[2] HungHChiangC: Estimation methods for time-dependent AUC models with survival data. *Canadian Journal of Statistics*.2009; 10.1002/cjs.10046 10.1002/cjs.10046

[3] UnoHCaiTTianLWeiL: Evaluating Prediction Rules fort -Year Survivors With Censored Regression Models. *Journal of the American Statistical Association*.2007;102(478) : 10.1198/016214507000000149 527-537 10.1198/016214507000000149

# Peer review: Referee response for version 1

**DOI:** 10.5256/f1000research.10162.r18159

**Published:** 2016-12-13

**Authors:** Motoki Shiga

**Affiliations:** 1 Department of Electrical, Electronic and Computer Engineering, Gifu University, Gifu, Japan

This paper proposed a weighted Cox proportional hazards model (DWCox) to reduce the effects of outliers. Experimental results demonstrated that DWCox outperforms the standard Cox model. The proposed method is interesting. This manuscript is well-written.

 Major comments:

1. Table 1 shows that the selected features by Halabi’s model (Halabi’s 8 features) and DWCox are quite different. A performance comparison the proposed model with a Cox model using Halabi’s 8 features would be a good demonstration of the proposed method.
2. Performance of DWCox and a standard Cox model were compared using only simulated data. A performance comparison using real datasets by leave-one-trial-out CV such as Table 4 is an important experiment to evaluate the proposed method.

 

 Minor comments:

1. p. 5 (in the above section of Eq. (2)): “sped up training” -> “speed up training”.

I have read this submission. I believe that I have an appropriate level of expertise to confirm that it is of an acceptable scientific standard.

# Response

**Published:** 2017-01-09

**Authors:** Jinfeng Xiao

**Affiliations:** University of Illinois at Urbana-Champaign, USA

Dear Dr. Shiga,

   Thank you for reviewing our manuscript! We appreciate your feedback. Here is our response to your major comments.

1. DWCox versus Halabi's model
  Halabi's model is the baseline method of the Prostate Cancer DREAM Challenge (PCDC). As described in the "Results on the PCDC data" subsection under the "Results" section, the better performance of DWCox compared to Halabi's model was validated by the challenge organizers using bootstrapping.
2. DWCox versus Cox in leave-one-trail-out cross-validation
  We tried both DWCox and a standard Cox in leave-one-trail-out cross-validations. The difference in iAUC is less than 1%, which is much smaller than the difference across the three leave-one-trial-out cross-validation experiments (Table 4). In this case the difference in iAUC is dominated by the inter-trial heterogeneity, and thus the contribution of density-based weighting is masked. It is also interesting that DWCox's iAUC (0.779) on the validation data set is much higher than its highest iAUC (0.685) in leave-one-trail-out cross-validation experiments. It indicates that the validation trial is better represented by the three training trials, compared to how well each training trial is represented by the other two.
